# Pulmonary function and exercise tolerance are related to disease severity in pre-dialytic patients with chronic kidney disease: a cross-sectional study

**DOI:** 10.1186/1471-2369-14-184

**Published:** 2013-09-04

**Authors:** Ruiter de Souza Faria, Natália Fernandes, Júlio César Moraes Lovisi, Maycon de Moura Reboredo, Murilo Sérgio de Moura Marta, Bruno do Valle Pinheiro, Marcus Gomes Bastos

**Affiliations:** 1 Interdisciplinary Nucleus of Study, Research and Treatment in Nephrology (NIEPEN), Federal University of Juiz de Fora, José Lourenço Kelmer, 1300, São Pedro, 36036-330 Juiz de Fora, Minas Gerais, Brazil; 2 Department of Medicine, Campus Universitário, Federal University of Juiz de Fora, Martelos, 36016-970 Juiz de Fora, Minas Gerais, Brazil; 3 Division of Pneumology, University Hospital of Federal University of Juiz de Fora, Rua Catulo Breviglieri, Santa Catarina, 36036110 Juiz de Fora, Minas Gerais, Brazil

**Keywords:** Respiratory function tests, Exercise tolerance, Physical fitness, Kidney failure chronic

## Abstract

**Background:**

Chronic kidney disease (CKD) involves a progressive, irreversible loss of kidney function. While early-stage CKD patients may show changes in pulmonary function and lowered exercise tolerance, the role of the estimated glomerular filtration rate (eGFR) in these patterns remains unknown. The aim of this study was to investigated pulmonary function and exercise tolerance in pre-dialytic CKD patients.

**Methods:**

A cross-sectional study was carried out with 38 adult volunteers divided into a control group (CG), consisting of 9 healthy adults, and 29 pre-dialytic CKD patients in stages 3 (G3), 4 (G4), and 5 (G5). All participants underwent spirometric and manovacuometric tests, a cardiopulmonary exercise test (CPET), a 6-minute walk test (6MWT), and laboratory tests.

**Results:**

The significant differences was observed in maximal exercise tolerance, measured as peak oxygen consumption percentage (VO_2_peak) (mL/kg/min) (CG = 28.9 ± 7.8, G3 = 23.3 ± 5.6, G4 = 21.4 ± 5.2, G5 = 20.2 ± 6.9; p = 0.03), and submaximal exercise tolerance, measured by 6MWT (m) (CG = 627.6 ± 37.8, G3 = 577.4 ± 66.1, G4 = 542.7 ± 57.3, G5 = 531.5 ± 84.2, p = 0.01). The eGFR was associated with pulmonary function-forced expiratory volume in the first second percentage (FEV_1_) (%) (r = 0.34, p = 0.02) and maximum inspiratory pressure (PImax) (r = 0.41, p = 0.02) - and exercise tolerance - VO_2_peak (mL/kg/min) (r = 0.43, p = 0.01) and 6MWT distance (m) (r = 0.55, p < 0.01).

**Conclusion:**

Pre-dialytic CKD patients showed lower maximal and submaximal exercise tolerances than healthy individuals.

## Background

Chronic kidney disease (CKD) is a worldwide public health problem. In Brazil, there are an estimated 2.9 million patients with an estimated glomerular filtration rate (eGFR) lower than 45 mL/min/1.73 m^2^, which classifies them as CKD stage 3B, 4 or 5 [1]. CKD often results in complications and comorbidities which compromise the function of various organs and may lead to premature mortality [2].

Respiratory problems are common in patients undergoing dialysis treatment, and these arise from a variety of factors, such as interstitial and alveolar edema, pleural effusion due to volume overload or increased capillary permeability [3], pulmonary hypertension [4], haemosiderosis [5], and weakness of the pulmonary muscles [6]. Haemodialysis patients present increased interstitial fluid volume, weakened muscles, and decreased diffusion capacity [7]. Peritoneal dialysis causes increased intra-abdominal pressure, which results in changes in respiratory mechanics [8]. A study of 109 patients receiving renal replacement therapy over 36 months showed an association between inflammation and worsened respiratory function, as well as a higher relative risk of mortality among patients with the worst forced vital capacity [9]. In fact, most of the available literature evaluated the pulmonary function in dialysis patients. However, the relation between the decrease in the eGFR and the respiratory function in pre-dialytic CKD patients remains unclear.

Various studies have shown that cardiovascular diseases, peripheral muscle dysfunction, anaemia and sedentary lifestyle result in a reduction in exercise tolerance and quality of life and are associated with higher mortality in CKD [10-13]. Renal transplant patients showed higher exercise tolerance than haemodialysis patients, which was attributed to improvement in kidney function associated with removal of uremic toxins [14]. Despite their potential relevance in CKD, exercise tolerance and pulmonary function have been little studied in pre-dialytic CKD patients [15] The aim of this study, therefore, was to evaluate the respiratory function and the exercise tolerance of pre-dialytic CKD patients in stages 3, 4, and 5.

## Methods

A cross-sectional study was performed from June through November 2011 as part of the Program of Secondary Prevention of Kidney Disease at the Interdisciplinary Nucleus of Studies, Research and Treatment in Nephrology of the Federal University of Juiz de Fora, Minas Gerais, Brazil.

The inclusion criteria were adult pre-dialytic CKD patients in stages 3, 4 and 5 based on the CKD staging proposed by the Kidney Disease Outcomes Quality Initiative [16]. The control group (CG) consisted of healthy individuals recruited from among the programme staff or their families. The study was approved by the Research Ethics Committee of the Federal University of Juiz de Fora and participants signed a consent form.

The exclusion criteria were as follows: patients older than 65 years; prior diagnoses of pulmonary diseases; current smokers, past smokers who quit less than 10 years before the study, or patients with a history of smoking more than 20 packs per year; cognitive or musculoskeletal conditions that would compromise test performance; unstable angina; an active infection in the previous 3 months; uncontrolled hypertension (systolic blood pressure ≥200 mmHg and/or diastolic blood pressure ≥ 120 mmHg); and the use of medication that could affect respiratory musculature function (e.g. steroids or cyclosporine).

Participants first underwent medical (anamnesis and physical examination) and physical (musculoskeletal) assessment to identify any clinical conditions that could limit their participation in the study. Immediately afterwards, blood samples were collected and tested for creatinine (mg/dL), potassium (mEq/L), haemoglobin (g/dL), calcium (mg/dL), phosphorus (mg/dL), albumin (g/dL), alkaline phosphatase (U/L), parathyroid hormone intact molecule (PTH-i) (pg/mL), total cholesterol (mg/dL), triglycerides (mg/dL), and venous gasometry. The eGRF was calculated from serum creatinine using Modification of Diet in Renal Disease equation [16].

The pulmonary function tests and functional capacity tests are exercise-dependent tests, and were therefore performed in different days so that the results would not be compromised because of fatigue in these tests. Evaluations were performed at 3 different visits. At first, the blood sample test and the 6-minute walk test (6MWT) were performed. In the second visit, the spirometry and the manovacuometry were performed with a interval of 1 hour between these tests. Cardiopulmonary exercise test (CPET) was performed only in the third visit. The visits were carried out between 15 to 30 days.

### Assessment of respiratory function

#### Spirometry

Patients underwent spirometry tests with a KoKo® apparatus (Koko Spirometer, Louisville, USA), following the recommendations of the Brazilian Society of Thoracic Medicine for tests of pulmonary function [17]. Before each test, the apparatus was calibrated with a 1 L syringe (Vitalograph Precision Syringe, Vitalograph, England), such that variability after 3 tests was < 3% (3 L). The forced expiratory volume in 1 second (FEV_1_), forced vital capacity (FVC), and the ratio FEV_1_/FVC were measured. From at least 2 sessions (with up to 8 attempts) the 2 highest values of FVC and FEV that differed by less than 0.15 L and peak expiratory flow (PEF) less than 10% or 0.5 L (whichever was greater) were selected. These values were standardised to percentages of expected values (%) based on data from the broader Brazilian population [18].

#### Manovacuometry

A Ger-ar® Classe B (São Paulo, Brazil) calibrated analog manovacuometer with an operational interval of ± 150 cmH_2_O was used to assess respiratory muscle strength via exercises to measure maximum inspiratory pressure (PImax) and maximum expiratory pressure (PEmax). Subjects were tested in a seated position, wearing a nose clip. Three measurements were taken with intervals of 2 seconds during which values were recorded. To compare results between groups, measured values converted to the expected percentage (%) were used for the broader Brazilian population [19].

#### Assessment of exercise tolerance

A CPET and a 6MWT were used to assess maximal and submaximal exercise tolerances, respectively.

#### Cardiopulmonary exercise test (CPET)

The CPET was carried out according to the recommendations of the American Thoracic Society [20] on an ergometric treadmill (Inbrasport®, Porto Alegre, Brazil) equipped with a computerised system for an exercise test (Ergo-PC Elite, Micromed Biotecnologia®, Brasília, Brazil). To analyse exhaled gases, patients wore a gas mask connected to a gas analyser (VO2000, Inbrasport®, Porto Alegre, Brazil). During the test, the electrocardiogram and heart rate were monitored continuously via 3 cutaneous electrodes placed to record the CM5 lead. In addition, blood pressure was monitored using the auscultatory method every 2 minutes. The ramp protocol was used, with constantly increasing incline which varied depending on the tolerance of each individual, until physical exhaustion was evident, despite being encouraged by the investigators, or until another criterion to interrupt the test was recorded. Peak oxygen consumption (VO_2_peak) was defined as the highest O_2_ consumption reached during the test. The values considered normal for VO_2_peak vary according to sex and age and are calculated using mathematical equations of the American College of Sports Medicine [21]. The anaerobic threshold (AT) was estimated using the V-slope method and ventilatory equivalents [22,23].

#### Six-minute walk test (6MWT)

The 6MWT was carried out according to the recommendations of the American Thoracic Society [24]. Individuals were instructed to walk as fast as possible during the 6 minutes on a flat 30-m track, and the distance walked was recorded in meters. Patients were allowed to stop and rest during the test but were instructed to resume walking as soon as they felt able to do so. The two tests were completed on the same day, with an interval of 30 minutes between each, and the farthest distance each patient walked was used to calculate the percentage relative to the predicted distance [25]. At the end of the test, perceived levels of effort were obtained using the modified Borg scale [26].

#### Statistical analysis

The descriptive analysis and the normality test (Shapiro Wilk) were performed. The descriptive statistics was used to explore patterns in the demographic, clinical, and laboratory variables, and in the variables that assess exercise tolerance and respiratory function. The data were expressed as means and standard deviations or percentages, depending on the distribution. Initially the group was divided in patients and controls and Student’s T test and Chi Squared were utilized for comparison. Subsequently, patients were divided into groups corresponding to CKD stages 3, 4 and 5 and compared with the control group. Among group comparisons were carried out with normally distributed data using ANOVA. For non-normally distributed data chi-squared test was used. The correlations between variables were tested with Pearson or Spearman’s correlation tests, based on the distribution of the variables. The significance level of p < 0.05 and a confidence interval of 95% were used. Analysis was carried out using SPSS 13.0 software.

## Results

**Figure 1:**
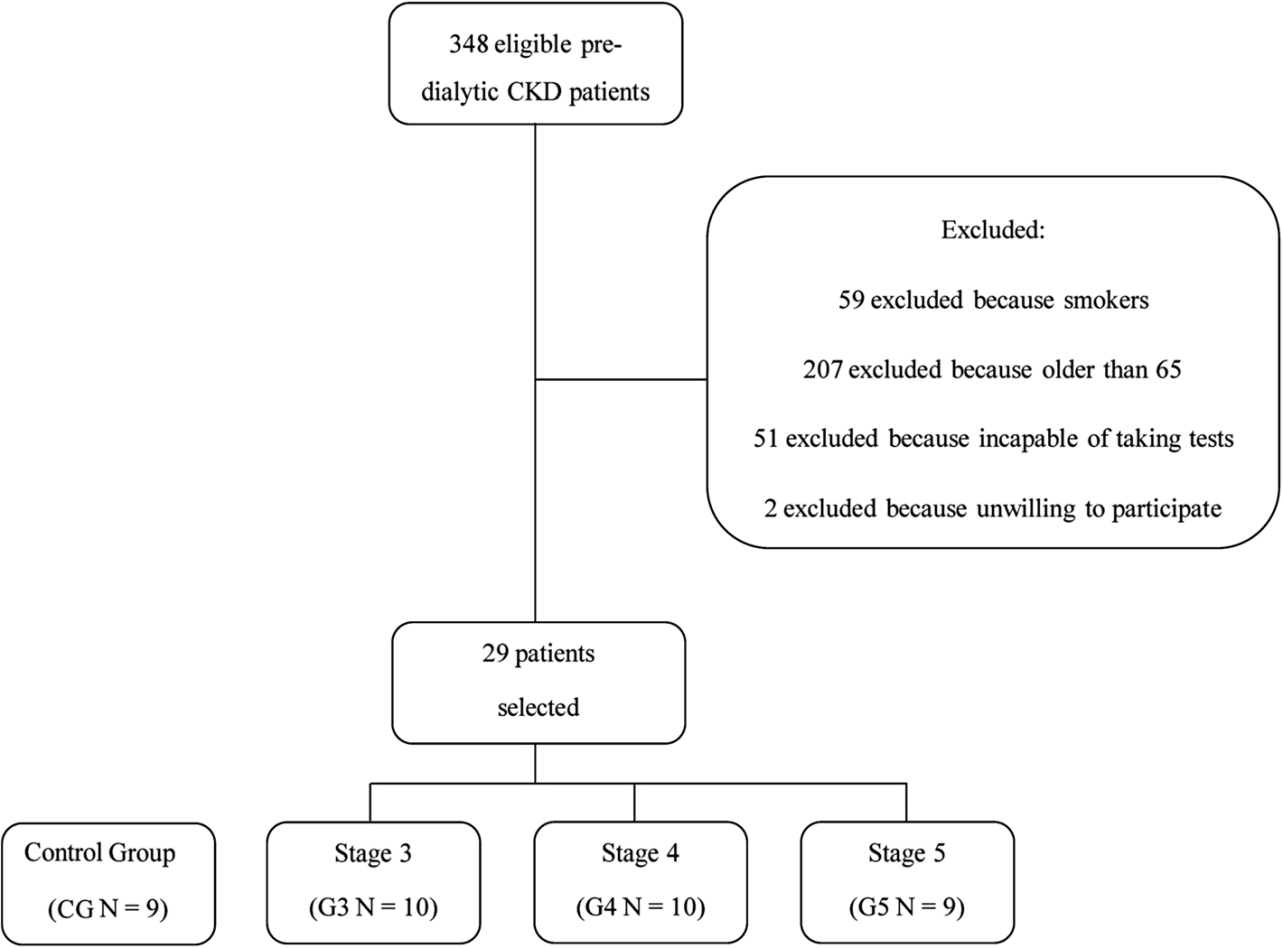
Flow diagram of selection process of study subjects.

Of the 348 pre-dialytic CKD patients assessed for eligibility, 317 were excluded from the study for the reasons shown in Figure 1. Twenty-nine of the 31 patients who fulfilled the criteria for inclusion in the study agreed to participate: 10 in CKD stage 3 (G3), 10 in stage 4 (G4) and 9 in stage 5 (G5). The control group consisted of 9 healthy individuals.

The mean time since diagnosis time was 4.47 ± 2.67 years (median 4 years). No statistical differences among groups in age, sex or body mass index were observed. The most prevalent cause of CKD was hypertensive nephropathy (34.4%), followed by chronic glomerulonephritis (20.6%) and diabetic nephropathy (17.2%). The most common comorbidities were hypertension (96.5%) and diabetes mellitus (24.1%). In the laboratory variables, there were significant differences in serum levels of potassium, alkaline phosphatase, PTH-i, haemoglobin, and bicarbonate (Table 1). Only 1 patient presented albumin levels of < 3.5 g/dL.

**Table 1:** Descriptive analysis of clinical and laboratory data

**Variables**	**Control**	**Stage 3**	**Stage 4**	**Stage 5**	**P**
	**(CG = 9)**	**(G3 = 10)**	**(G$ = 10)**	**(G5 = 9)**	
Age (years)	51.5 ± 7.5	56.8 ± 5.8	52.3 ± 8.5	56.3 ± 9.0	0.351
Sex (F/M)	4/5	5/5	6/4	5/4	0.914
BMI (kg/m^2^)	27.2 ±4.6	29.5 ± 5.7	26.3 ± 6.5	27.9 ± 5.6	0.655
CKD Etiology:					
Hypertensive Nx	-	5	3	2	<0.001*
Diabetic Nx	-	1	2	2	
CGN	-	1	2	3	
Others	-	3	3	2	
Comorbidities:					
H	-	10	9	9	<0.001*
DM	-	2	4	2	<0.001*
Dyslipidemia	-	6	6	8	<0.001*
eGFR (mL/min/1.73m^2^)	77.6 ± 9.8^b,c,d^	43.8 ± 8.7^a,c,d^	21.3 ± 4.2^a,b,d^	12.7 ± 3.7^a,b,c^	<0.001*
Creatinine (mg/dL)	0.9 ± 0.1^b,c,d^	1.5 ± 0.2^a,c,d^	2.7 ± 0.6^a,b,d^	4.4 ± 1.3^a,b,c^	<0.001*
Potassium (meq/L)	4.6 ± 0.4^d^	4.4 ± 0.5^d^	4.7 ± 0.9	5.4 ± 0.9	0.049
Calcium (mg/dL)	10.0 ± 0.6	9.9 ± 1.0	9.9 ± 0.7	10.2 ± 0.6	0.852
Phosphorus (mg/dL)	4.8 ± 2.2	3.6 ± 0.6	4.1 ± 0.8	4.9 ± 0.6	0.081
Alkaline phosphatase (U/L)	155.2 ± 41.1^d^	193.8 ± 38.3^d^	220.1 ± 51.1^d^	305.5 ± 144.7	0.014
PTHi (pg/mL)	71.2 ± 29.8^d^	77.0 ± 24.9^d^	114.2 ± 52.2^d^	337.2 ± 283.0	0.004
Hemoglobin (g/dL)	15.0 ± 1.8^c,d^	13.7 ± 1.4^c^	12.3 ± 1.3	12.3 ± 1.6	0.003
Albumin (g/dL)	3.9 ± 0.1	4.2 ± 0.3	3.6 ± 0.5	4.2 ± 0.5	0.102
Total cholesterol (mg/dL)	201.6 ± 41.5	207.8 ± 43.5	190.6 ± 40.7	215.4 ± 59.3	0.698
Triglycerides (mg/dL)	106.5 ± 45.9	147.6 ± 81.8	179.1 ± 120.0	252.1 ± 221.3	0.163
HCO_3_ (mmol/L)	28.0 ± 0.8^c,d^	27.3 ± 2.5^c,d^	23.7 ± 3.8	22.0 ± 4.5	0.003

<0.001* = P-value for comparison between the control Group and the CKD groups (Stage 3,4 and 5); *BMI* Body Mass Index, *Nx* Nephropathy, *CKD* Chronic kidney disease, *CGN* Chronic glomerulonephritis, *H* Hypertension, *DM* Diabetes Mellitus, *eGFR* estimated Glomerular Filtration Rate, *PTHi* Parathyriod Hormone Intact Molecule, *HCO*_*3*_ Bicarbonate.

Post hoe analysis: a = differs from CG at p<0.05; c = differs from G4 at p<0.05; d = differs from G5 at p<0.05.

In this study, only 2 (6.8%) patients were anaemic, and these were incidents in the clinic and they had not yet reversed this condition, and used erythropoietin. About 37.9% of the patients used beta-blockers and all hypertensive patients were using ACE inhibitors and/or angiotensin receptor blockers. Sodium bicarbonate, calcium chelating and vitamin D agents were used where necessary, in accordance with the guidelines of the Brazilian Society of Nephrology [27,28].

There were no significant differences in spirometric measures among groups. Among the manovacuometry variables, PImax (%) differed between groups, but the difference was not statistically significant (p = 0.06) (Table 2).

**Table 2:** Assessment of pulmonary function (spirometry and manovacuometry)

**Variables**	**Control**	**Stage 3**	**Stage 4**	**Stage 5**	**P**
	**(CG = 9)**	**(G3 = 10)**	**(G4 = 10)**	**(G5 = 9)**	
**Spirometry**					
FVC (%)	118.0 ± 136.7	101.7 ± 20.2	98.3 ± 15.1	97.3 ± 21.1	0.115
FEV, (%)	111.0 ± 10.4	100.4 ± 18.4	95.0 ± 13.1	92.1 ± 19.1	0.126
FEV,/FVC (%)	94.9 ± 4.5	99.3 ± 7.8	97.5 = 5.3	95.0 ± 4.0	0.333
Manovacuometry					
PImax (%)	93.3 ± 14.3	63.2 ± 27.1	62.0 = 12.5	69.9 ± 29.0	0.061
PEmax (%)	108.4 ± 16.5	94.1 ± 21.9	84.4 = 16.4	89.6 ± 25.6	0.192

*FVC* Forced Vital Capacity, *FEV*_*1*_ Forced Expiratory Volume in the first second of forceful exhalation, *PImax* Maximum Inspiratory Pressure, *PEmax* Maximum Expiratory Pressure.

**Figure 2:**
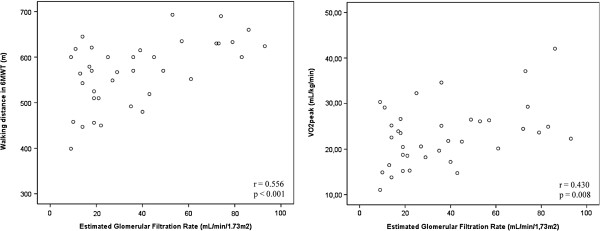
Correlations between estimated glomerular filtration rate and six-minute walk test (6MWT) and cardiopulmonary exercise test (CPET).

In the CPET, VO_2_peak was lower in the G4 and G5 groups than in the CG (p = 0.03), while values of relative power maximal (RPmax) (p < 0.01), oxygen consumption at anaerobic threshold (VO_2_AT) (p < 0.01) and maximum heart rate (HRmax) (p < 0.01) were lower in the 3 CKD groups than in the control. Likewise, distance walked in the 6MWT was lower in the G4 and G5 groups than in the CG (Table 3). Maximal exercise tolerance (VO_2_peak and RPmax) and submaximal exercise tolerance (VO_2_AT and 6MWT), FEV_1_ and FCV were correlated with eGFR (Table 4 and Figure 2). The criteria for discontinuation of CPET were the same as recommended by the American Thoracic Society [20] however in all tests there were no complications; in addition, all tests were stopped when the patients reached physical exhaustion.

**Table 3:** Assessment of exercise tolerance (cardiopulmonary exercise test and six-minute walk test)

**Variables**	**Control**	**Stage 3**	**Stage 4**	**Stage 5**	**P**
	**(CG = 9)**	**(G3 = 10)**	**(G4 = 10)**	**(G5 = 9)**	
Cardiopulmonary test					
RPmax (W/kg)	4.9 ± 1.3	3.7 ± 0.9^a^	3.0 ± 1.2^a^	3.2 ± 1.0^a^	0.007
VO_2_peak (mL/kg/min)	28.9 ± 7.8	23.3 ± 5.6	21.4 ± 5.2^a^	20.2 ± 6.9^a^	0.033
VO_2_peak (%)	85.4 ± 18.2	75.8 ± 18.3	65.9 ± 16.0^a^	63.4 ± 16.0^a^	0.038
VO_2_AT (mL/kg/min)	20.5 ± 4.1	15.7 ± 3.5^a^	16.6 ± 3.8^a^	13.6 ± 3.8^a^	0.005
VEmax (l/min)	68.2 ± 20.3	60.8 ± 20	49.7 ± 20	56.9 ± 28.9	0.356
HRmax (bpm)	165 ± 14	146 ± 16^a^	139 ± 23^a^	129 ± 18^a^	0.003
HRmax (%)	97.9 ± 34.6	90.0 ± 11.1	82.8 ± 11.5^a^	79.3 ± 9.9^a^	0.003
SBPmax (mmHg)	197.5 ± 34.6	202.6 ± 25.8	204.2 ± 35.5	201.7 ± 34.3	0.976
DPBmax (mmHg)	89.7 ± 7.5	97.6 ± 9.2	101.8 ± 18.7	98.8 ± 9.6	0.203
6MWT					
Distance (m)	627.6 ± 37.8	577.4 ± 66.1	542 ± 57.3^a^	531.5 ± 84.2^a^	0.012
% Predicted (%)	90.5 ± 7.6	93.9 ± 7.5	83.4 ± 9.5^b^	83.0 ± 8.9^b^	0.017

*RPmax* Maximum Relative Power, *VO*_*2*_*peak* Peak Oxygen Consumption, *VO*_*2*_*AT* Oxygen Consumption at Anaerobic Threshold, *VE* Minute Ventalation, *HRmax* Maximum Heart Rate, *SBPmax* Maximum Systolic Blood Pressure, *DBPmax* Maximum Diastolic Blood Pressure.

Post hoc analysis: a = differs from CG at p<0.05; b = differs from G3 at p<0.05.

**Table 4:** Correlations between the estimated glomerular filtration rate, spirometric variables, and exercise tolerance variables

**Variables**	**Estimated glomerular filtration rate (mL/min/1.73 m**^**2**^**)**	
	**r**	**p**
FVC (%)	0.348	0.041
FEV, (%)	0.349	0.020
RPmax (W/kg)	0.536	0.001
VO_2_peak (mL/kg/min)	0.430	0.008
VO_2_AT (mL/kg/min)	0.481	0.003
6MWT (m)	0.556	< 0.001

The correlation between pulmonary function and exercise tolerance was performed and a weak correlation between FVC (%) and VO_2_peak: r = 0.34 and p = 0.05; FVC (%) and VO_2_peak (%): r = 0.37 and p = 0.03; and FEV_1_ (%) and VO_2_peak (%): r = 0.37 and p = 0.03 were observed.

## Discussion

In this study was observed that pre-dialytic CKD patients showed lower maximal and submaximal exercise tolerances than healthy individuals. In addition, eGFR was correlated with reduced exercise tolerance and pulmonary function.

Most studies of respiratory function and exercise tolerance in CKD patients have included patients undergoing haemodialysis or peritoneal dialysis, treatments that can interfere with pulmonary function [7,8]. The few studies performed in pre-dialytic CKD patients have not excluded factors that may contribute to reduced exercise tolerance and impaired pulmonary function, such as smoking [11] or were carried out with children [29]. Because major confounders such as aging, smoking, previous pulmonary disease and medications that can interfere with respiratory function were excluded, this study was able to assess only the impact of CKD and its associated changes in exercise tolerance and lung function.

So far, few studies have been performed on exercise tolerance in patients not yet on dialysis [15]. In CKD patients, particularly those undergoing dialysis, reduced exercise tolerance is associated with conditions such as anaemia, sedentary lifestyle, decreased muscular strength and resistance, and chronic inflammation [10,30-32]. Exercise tolerance can be assessed by relatively complex tests such as CPET or by simple low-cost tests such as 6MWT.

The incremental CPET quantifies VO_2_peak, which is considered the gold standard for determining maximal exercise tolerance. In a recent review, Johansen and Painter [33] report a mean reduction of 50% - 80% of VO_2_peak in pre-dialytic CKD patients. In this study, VO_2_peak was also lower in pre-dialytic CKD patients than in the CG and correlated with eGFR. Accordingly, in a study published by Clyne et al. [15], the authors showed in pre-dialytic CKD patients, that exercise tolerance reduces with progress of kidney disease and VO_2_peak of these patients is approximately 60% lower than healthy controls. The CPET also offers important information on submaximal exercise tolerance by determining metabolic responses at the AT, which permit an assessment of the efficiency of aerobic metabolism. During activities performed below the AT, energy sources are aerobic, and there is no sustained accumulation of lactate. On the other hand, anaerobic glycolysis occurs above this threshold, which results in the production and accumulation of lactate [34]. In the present study, reduced VO_2_AT, indicative of less efficient energy production by aerobic mechanisms, was observed in patients with lower eGFR. Clinically, the less efficient aerobic mechanism leads to greater muscular fatigue and lower exercise tolerance.

Submaximal exercise tolerance may also be quantified by the 6MWT. This test is easy to perform, requires no special equipment, is better tolerated by patients and is more representative of daily activities than the incremental CPET [24]. Some authors have reported lower distances in 6MWT in CKD patients when compared with healthy individuals [35-37]. In one such study, Cury et al. [37] associated the reduction in functional capacity with inefficiencies in the uptake, transport, and use of O_2_ caused by dysfunction of cardiovascular, respiratory and muscular systems.

In the present study, also was recorded shorter distances walked in the 6MWT by CKD patients and this result was more evident in those with lowest eGFR. Since the ventilatory response (minute ventilation) was not altered during the CPET in CKD patients, the decreasing in exercise tolerance in these patients can be associated with disturbance in cardiovascular system or peripheral muscles. In addition, pulmonary function was assessed by spirometry and showed no differences between groups, supporting the findings obtained in the CPET. Moreover, there was only a weak correlation between pulmonary function and exercise tolerance.

The reduced exercise capacity in patients on haemodialysis may be due to changes in transport mechanisms and oxygen extraction. The transport of oxygen in these patients may be altered by reduced cardiac output, changes in maximum heart rate and decreased arterial oxygen by anemia, while the impairment of oxygen extraction may be due to uremic myopathy and disuse atrophy [38-40]. In this study, the hemoglobin level was appropriate and not correlated with lung function and CPET related variables.

CKD patients may present conditions that result in ventilatory limitations, such as musculoskeletal weakness [37,41,42], interstitial edema and edema of small airways [13], pleural effusion [17] and osteoarticular changes in the thoracic vertebrae [43]. In the present study, there were no significant differences in spirometric test results between CKD patients and the CG, but a positive correlation between eGFR, FVC, and FEV_1_ was observed, which suggests some decline in pulmonary function associated with deteriorating kidney function. The relatively small number of patients that was studied and the small magnitude of the loss of pulmonary function may explain the lack of statistically significant differences between groups in the spirometric data.

Decreased muscular strength may have multiple causes, including reduced carnitine level, hypovitaminosis D, hypotrophy of type-II muscle fibres, decreased energy use by muscle fibres, increased PTH-i [29], metabolic acidosis, chronic inflammation [44], decreases in oxidative metabolism, decreases in serum levels of calcium and increases in protein catabolism [37]. In the present study, CKD patients showed a tendency of lower values of PImax (%) than the CG (p = 0.06), in agreement with previous reports that skeletal muscles, including the respiratory musculature, presented lower strength and resistance over the course of CKD [9,22]. It is also possible that changes to other musculoskeletal groups, such as those in the lower limbs, also contributed to decreased exercise tolerance.

This study presented limitations. First, the fact that it is a cross-sectional study limits the interpretation of the impact of CKD on the observed results. Second, the exclusion of possible factors known to decrease pulmonary function limited the size of study sample. Third, these results cannot be interpreted as broadly representative, because the study was carried out in non-elderly patients at a single centre. Finally, it is important to emphasize that the patients who participated in this study were also participating in a CKD secondary prevention programme, received interdisciplinary treatment and were clinically stable and under close observation.

## Conclusion

The present study showed that pre-dialytic CKD patients had a reduction in maximal and submaximal exercise tolerances. In this context, CPET can be used for exercise prescription before an inclusion in exercise programmes which should be started early in the course of the disease. Nevertheless, further studies are necessary to confirm these outcomes.

## Abbreviations

(CKD): Chronic kidney disease; (eGFR): Estimated glomerular filtration rate; (CG): Control group; (G3): Stage 3; (G4): Stage 4; (G5): Stage 5; (CPET): Cardiopulmonary exercise test; (6MWT): 6-minute walk test; (VO2peak) (%): Peak oxygen consumption percentage; (FEV1) (%): Forced expiratory volume in the first second percentage; (PImax): Maximum inspiratory pressure; (PTH-i): Parathyroid hormone intact molecule; (FVC): Forced vital capacity; (PEF): Peak expiratory flow; (PEmax): Maximum expiratory pressure; (VO2max): Peak oxygen consumption maximum; (AT): Anaerobic threshold; (RPmax): Relative power maximum; (VO2AT): Oxygen consumption at anaerobic threshold; (HRmax): Maximum heart rate.

## Competing interests

The authors declare the following non-financial competing interests.

## Authors’ contributions

All the authors of this manuscript made substantial contributions to the conception and design, acquisition of data, and analysis and interpretation of data. All authors read and approved the final manuscript.

## Pre-publication history

The pre-publication history for this paper can be accessed here:

http://www.biomedcentral.com/1471-2369/14/184/prepub
